# New Approach of Anti-VEGF Agents for Age-Related Macular Degeneration

**DOI:** 10.1155/2012/637316

**Published:** 2012-02-09

**Authors:** Young Gun Park, Hyun Wook Rhu, Seungbum Kang, Young Jung Roh

**Affiliations:** Department of Ophthalmology, Yeouido St. Mary's Hospital, College of Medicine, The Catholic University of Korea, No. 62 Yeouido-dong, Yeongdeungpo-gu, Seoul 150-713, Republic of Korea

## Abstract

Age-related macular degeneration (AMD) is the leading cause of visual loss in older population. Angiogenesis is an important factor associated with the development of CNV due to AMD. Treatment of CNV with intravitreal anti-VEGF monotherapy is currently the standard of care. However, not all patients respond to monotherapy, and modified anti-VEGF treatment regimen and combination therapy may target reducing treatment frequency or improving visual outcome. This paper reviews the many clinical trials that have been performed utilizing several treatment regimens. While many trials have shown that this variable therapy is justifiable, further study is required to determine correct regimens and dosage.

## 1. Introduction

Age-related macular degeneration (AMD) is one of the leading causes of substantial and irreversible vision loss. The prevalence of AMD can be expected to increase along with life expectancy, which has risen steadily [1, 2]. Without treatment, the neovascular form of AMD leads to severe quality-of-life loss within a short time period and considerable economic burden. 

VEGF is a key mediator involved in the control of angiogenesis and vascular permeability and has been shown to be induced by hypoxia in cultured human RPE [3]. Vascular endothelial growth factor A (VEGF-A) is the most potent promoter of angiogenesis and vascular permeability within the VEGF family and its role in the pathogenesis of neovascular AMD is well recognized [4, 5]. The advent of intravitreous VEGF inhibitors has revolutionized the management of neovascular AMD. Yet, frequently, indefinite injections of VEGF blocking agents introduce a significant treatment burden for patients with neovascular AMD. Many studies on modified treatment regimens have been performed in an attempt to mitigate this burden without compromise to visual acuity outcomes. Meanwhile, various randomized clinical trials on combination therapies and efforts to develop new pharmacologic agents are ongoing.

## 2. Material and Methods

A MEDLINE search of the English language literature from 1990 to present was conducted. The search strategy was based on combinations of medical subject headings (MeSH) and keywords and was not restricted to specific journals or years of publication. The searches were supplemented by handsearching the bibliographies of included studies and reviews.

## 3. Results

### 3.1. Three Antivascular Endothelial Growth Factor (VEGF) Therapies

Three antivascular endothelial growth factor (VEGF) therapies are currently used for the treatment of patients with wet age-related macular degeneration (AMD): pegaptanib (Macugen, Pfizer, UK), ranibizumab (Lucentis, Novartis, UK), and bevacizumab (Avastin, Roche, UK). 

Petaganib is an oligonucleotide aptamer and was the first VEGF antagonist to be approved by the US Food and Drug Administration for use in wet AMD. However, wet AMD patients treated with petaganib still experience visual decline [2, 6]. For this reason, petaganib was seldom used now.

Ranibizumab (Lucentis) is also a humanized antibody fragment against VEGF which was specifically designed for intraocular use as a smaller antibody fragment to penetrate through the retina better. The Food and Drug Administration (FDA) approved ranibizumab for treatment of subfoveal neovascular AMD in June, 2006. It was the first treatment for AMD shown to improve visual acuity in a substantial percentage of patients. 

Bevacizumab (Avastin) is a recombinant humanized monoclonal immunoglobulin antibody that inhibits the activity of VEGF. It has a similar action and is related to the ranibizumab compound with respect to its structure. Bevacizumab was approved by the FDA for the treatment of metastatic colorectal cancer in 2004, but it has not been licensed for the treatment of wet AMD or any other ocular conditions. However, it is recently used off-label worldwide not only for wet AMD but also for other ocular disease entities associated with macular edema and abnormal vessel growth. 

Since 2009, there have been increasing number of studies that have compared the properties of ranibizumab and bevacizumab and investigated their efficacy on AMD. The pivotal phase III Minimally Classic/Occult Trial of the Anti-VEGF Antibody Ranibizumab in the Treatment of Neovascular AMD (MARINA) [7] and the Anti-VEGF Antibody for the Treatment of Predominantly Classic CNV in AMD (ANCHOR) trial [8, 9] demonstrated best-corrected visual acuity (BCVA) outcomes far superior to any previously published study in the treatment of this disease. At the end of 24 months in the MARINA trial, significantly more ranibizumab-treated patients had maintained (lost <15 Early Treatment Diabetic Retinopathy Study (ETDRS) letters) or improved vision than sham-injected patients. Indeed, 90–95% of patients treated with 0.3 and 0.5 mg ranibizumab maintained vision compared with 53–64% of control patients. Over the same period, vision improved in 25–34% of treated eyes, compared with 4-5% of sham-injected patients. 

In the ANCHOR trial, ranibizumab was compared with verteporfin photodynamic therapy (PDT) and demonstrated similar findings: 90–96% of the ranibizumab-treated versus 64–66% of the PDT-treated patients maintained vision, whereas 34–41% versus 6% of each group, respectively, gained more than 15 letters. These outcomes were significantly better (*P* < 0.001) than those achieved by the control groups.

Patient-reported outcomes were also assessed in the ANCHOR and MARINA trials to measure the influence of the ranibizumab-mediated improvement in VA on quality of life. The data demonstrated that patients treated with ranibizumab were more likely to report improvements in near activities, distance activities, and vision-specific dependency which were maintained over the 2-year duration of the trial [10, 11]. These data demonstrate that the clinical improvements seen with ranibizumab treatment translate into meaningful benefits for the patient. 

Bevacizumab, the predecessor of ranibizumab, is a full-length monoclonal antibody that binds to and blocks the action of all VEGF isoforms. Numerous retrospective [12–15] and prospective studies [16–18] of intravitreal bevacizumab have reported its efficacy for neovascular AMD and low rates of treatment-related complications [19]. Although a number of these studies were uncontrolled, relatively small in sample size, of limited followup, and varied with regard to outcome measures and retreatment criteria, the reported efficacy of bevacizumab coupled with its low cost when utilized as an intraocular agent has propelled its adoption worldwide. 

In clinical practice, many retinal physicians have extrapolated the data and continued using bevacizumab. A formal head-to-head comparison of bevacizumab and ranibizumab is being conducted by the National Eye Institute of the National Institute of Health in the Comparisons of Age-Related Macular Degeneration Treatment Trials (CATTs) [20, 21]. The CATT study design includes four treatment arms: either bevacizumab or ranibizumab on a variable schedule and either bevacizumab or ranibizumab on a fixed monthly schedule for 1 year followed by random assignment to either continued monthly injections or a variable schedule based on the treatment response. The primary outcome measure is mean change in BCVA; secondary outcome measures include number of treatments, anatomical changes in the retina, adverse events, and cost. Preliminary results are reported in 2011 and will provide insight into how ranibizumab and bevacizumab compare with each other within the context of either a fixed monthly or traditional pro re nata (PRN) approach. At 1 year, bevacizumab and ranibizumab had equivalent effects on visual acuity when administered according to the same schedule. Bevacizumab administered monthly was equivalent to ranibizumab administered monthly, with 8.0 and 8.5 letters gained, respectively. Bevacizumab administered as needed was equivalent to ranibizumab as needed, with 5.9 and 6.8 letters gained, respectively. Ranibizumab given as needed with monthly evaluation had effects on vision that were equivalent to those of ranibizumab administered monthly, although the comparison between bevacizumab as needed and monthly bevacizumab was inclusive. Differences in rated of serious adverse events require further study.

### 3.2. Modified Treatment Regimens

The prospect of indefinitely adhering to the monthly treatment schedules of MARINA and ANCHOR has raised ocular and systemic safety concerns as well as convenience and cost issues for patient and physician alike. The identification of alternative dosing strategies capable of reducing the number of required anti-VEGF injections while still achieving visual acuity outcomes similar to those reached in the pivotal trials has since been a subject of great interest. 

**Figure 1:**
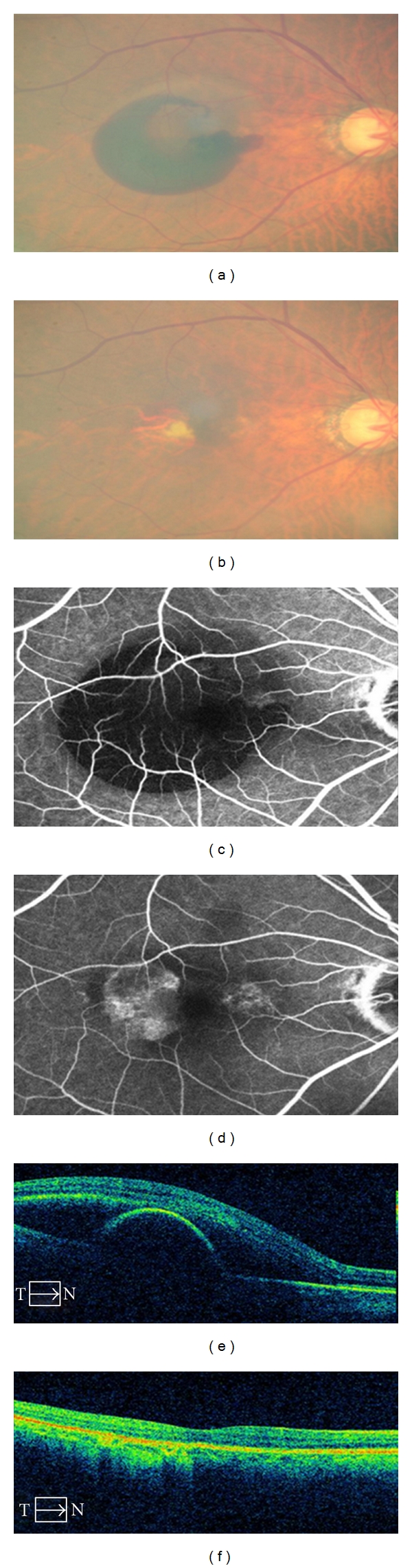
Fundus photograph of patients with hemorrhagic PED secondary to AMD. Fluorescein angiogram shows large hypofluorescence due to hemorrhage at macular lesion. Optical coherence tomography with large PED and subretinal fluid. (a, c, e) Same section after 3 ranibizumab intravitreal injections. Complete resolution of the hemorrhagic PED with edema is illustrated. There was also improvement in visual acuity (b, d, f).

The observed biphasic treatment effect raised the possibility that, after the initial 3-month loading phase, maintenance of VA gain may be achieved with less frequent treatments. A PIER trial evaluated ranibizumab administered monthly for 3 months, followed by quarterly injections, and compared this with sham treatment. Under this schedule, ranibizumab did provide a significant VA benefit; a significantly greater number of patients achieved VA stabilization at 24 months compared with patients receiving sham treatment. However, subgroup analysis revealed that VA gains observed during the first 3 months of treatment were only maintained in 40% of patients over the duration of the trial, and for the remaining 60% quarterly dosing was not suitable [22, 23]. Results for both ranibizumab doses in the PIER trial (0.3 and 0.5 mg) showed an initial mean improvement in BCVA during the initiation phase with monthly dosing, but after month 3 in the maintenance phase with quarterly dosing, there was a gradual decline in mean BCVA to below the pretreatment baseline (_2.2 letters) at 12 months, which remained unchanged at 24 months [23] (Figure 1). 

More recently, the Efficacy and Safety of Ranibizumab in Patients with Subfoveal Choroidal Neovascularization Secondary to Age-Related Macular Degeneration (EXCITE) study directly compared the PIER regimen with a fixed monthly treatment arm (0.3 mg ranibizumab) [24]. Although BCVA outcomes in the two quarterly treatment arms fared better than those in the PIER study at 12 months (2.2 and 3.1 letters with 0.3 and 0.5 mg ranibizumab, resp.), neither was as good as monthly dosing (0.9 letters). These suboptimal results demonstrate that, on average, quarterly treatment is inferior to monthly treatment; thus, it has never been adopted in practice. Subsequent to the PIER trial, further investigation of a flexible dosing approach was carried out. The EXCITE trial directly compared a maintenance phase of quarterly injections against the monthly regimen. Consistent with previous observations, an initial gain was made in the first 3 months, after which patients receiving monthly injections contributed to gain VA, whilst those receiving quarterly injections showed a decrease from their 3-month VA levels.

The current norm in clinical practice with ranibizumab or bevacizumab is to implement an initiation/induction phase followed by an individualized maintenance phase that is modeled after one of two basic approaches: traditional PRN [25] or “treat and extend” [26, 27]. Traditional PRN involves both regular followup and treatment until the macula is more or less free of exudation, with treatment thereafter during the maintenance phase only in the presence of recurrent exudation. The original prospective studies that evaluated a PRN approach to the maintenance phase were the Prospective Optical Coherence Tomography Imaging of Patients with Neovascular AMD Treated with Intra-Ocular Lucentis (PrONTO) study [28] and the Secondary to Age-Related Macular Degeneration (SAILOR) study [29]. More recently, the Study of Ranibizumab in Patients with Subfoveal Choroidal Neovascularization Secondary to Age-Related Macular Degeneration (SUSTAIN) study has contributed additional data [30]. In each of these trials, patients received three consecutive, monthly intravitreal injections of ranibizumab for induction, followed by monthly office visits. Thereafter, a PRN maintenance phase adhered to the following retreatment criteria: loss of at least five ETDRS letters, increase in central macular thickness on OCT of at least 100 *μ*m, or new hemorrhage. 

Of the three studies, the PrONTO study demonstrated the best visual acuity results. The PrONTO study evaluated an OCT guided, variable-dosing regimen with ranibizumab (0.5 mg) and showed that mean visual acuity improved by 9.3 ETDRS letters at 12 months. Over a 2-year period, mean BCVA outcomes were similar to MARINA and ANCHOR with a mean of 9.9 injections (5.6 in the first year and 4.3 in the second). In comparison, results from the SAILOR study were not as good. In this study, the mean change in BCVA at 12 months from baseline was 0.5 and 1.7 letters in the treatment-naive and previously treated groups, respectively, at the 0.3 mg dose and 2.3 letters in both groups at 0.5 mg. It is worth noting that participants were not monitored as closely in SAILOR as compared with PrONTO, averaging nine visits through 1 year and a mean of 4.9 injections. 

The 12-month results from SUSTAIN were slightly better than those from SAILOR (mean BCVA from baseline of 3.6 letters), yet still not as good as the monthly treatment trials. In contrast to SAILOR, participants in the SUSTAIN trial were followed monthly (more like PrONTO) and the mean number of injections over the first year was higher at 5.6. 

Other relatively large studies using a traditional PRN approach have recently been published [31–33]. An analysis of these reports highlights an important trend: the best visual acuity results come from the study with the greatest mean number of treatments and closest followup, whereas the poorest outcomes were observed in the study with the lowest mean number of treatments and office visits. Unlike traditional PRN, a treat and extend approach initially involves regular and frequent treatment until the macula is dry, followed by a gradual extension of the treatment interval and corresponding followup visit. Treatment interval extension continues until there are signs of recurrence, at which point the treatment interval is then reduced. 

Kang and Roh [34, 35] recently published a retrospective analysis that monthly injections were not given in contrast to the three injections during the initial treatment period in the PIER and PrONTO trials. This study minimized the number of injections given during 12 months of follow-up (a mean of 4.07 injections were given over the 12 months). The decreased need for retreatment is of great benefit to both patients and clinicians. These results may raise doubts about the need for the three initial loading injections. They reported another study [35]; the mean number of injections given in the 12 months period was 4.2 (range, 1–6). Patients were also offered reinjection with ranibizumab on an “as needed” basis. Data showed that the percentage of patients (71.9%) with no visual loss or improved visual acuity was comparable to the percentages in the monthly injection-based studies. 

In addition, Gupta et al. evaluated a treat and extend approach with bevacizumab and found nearly identical results at 12 months following a mean of 7.3 injections in the first year [33]. Although various methods for individualizing maintenance therapy have been proposed, the optimal nonmonthly dosing regimen does not remain clear.

### 3.3. Combination Therapy: Photodynamic Therapy and Anti-VEGF Therapy

The development and propagation of CNV membranes involve proangiogenic factors, vascular permeability molecules, and inflammatory proteins. Current standard treatment with monthly intravitreal injections of anti-VEGF monotherapy can be limited to the angiogenic component of CNV development and burdensome for both the physician and patient. Patients are subjected to increased risk with monthly treatments that may be lessened with treatment options given with less frequency [36]. Combination therapy with PDT proven to be effective may not only have a role in the treatment of CNV development but also may provide synergy through blocking adverse effects. 

Photodynamic therapy was approved in 2000 by the FDA for the treatment of CNV secondary to AMD. Treatment involves intravenous administration of a light-sensitive dye called verteporfin followed by laser-guided, location-specific activation within the CNV membrane. Activation of the verteporfin molecules incites a phototoxic event within blood vessels, induces endothelial cell damage, platelet aggregation, and eventually leads to thrombosis of vascular channels. Treatment size is limited by the greatest linear diameter of the CNV lesion being treated [37, 38]. 

While PDT is intended to specifically target CNV vessels, collateral damage to surrounding blood vessels may lead to ischemia of healthy tissue. Following PDT of a CNV membrane, induced ischemia can lead to production of proangiogenic factors, especially VEGF. Therefore, combining verteporfin PDT and anti-VEGF therapy may be beneficial compared with either modality alone, yielding longer treatment-free intervals and requiring fewer intravitreal injections [37]. 

The RhuFab V2 Ocular Treatment Combining the Use of Visudyne to Evaluate Safety (FOCUS) study is a multicenter, randomized, single-blind study designed to evaluate the safety and efficacy of standard fluence Photodynamic therapy (sfPDT) in combination with intravitreal ranibizumab [39, 40]. It compared sfPDT to combination sfPDT and intravitreal ranibizumab in the treatment of predominantly classic CNV secondary to AMD. One-year data showed greater visual stability in the patients treated with combination therapy and 23.8% of patients experienced improvement in visual acuity, compared with 5% of patients treated with PDT monotherapy alone. The number of retreatments with sfPDT was decreased as well with 91% of patients treated with sfPDT monotherapy requiring repeat treatment while only 28% of patients treated with combination therapy requiring retreatment. Two-year data showed similar results with 88% of combination-treated patients losing less than 15 lines of vision versus 75% of sfPDT-alone treated patients. Combination therapy required an average of 0.4 repeat PDT treatments compared with an average of 3.0 in the sfPDT group.

### 3.4. Vascular Endothelial Growth Factor Trap-Eye

The most effective dosing regimen and monitoring program for anti-VEGF therapy has yet to be firmly established but new treatments are aimed at extending and improving on the efficacy of ranibizumab. VEGF Trap-Eye is a promising new anti-VEGF drug (aflibercept ophthalmic solution; Regeneron Pharmaceuticals Inc., Tarrytown, NY, USA). Structurally, VEGF Trap-Eye is a fusion protein of key binding domains of human VEGFR-1 and -2 combined with a human IgG Fc fragment. Functionally, VEGF Trap-Eye acts as a receptor decoy with high affinity for all VEGF isoforms, binding more tightly. VEGF Trap-Eye differs from established anti-VEGF therapies in its higher binding affinity for VEGF-A and its blockage of placental growth factors-1 and -2 [41, 42]. 

Recently, the 1-year results of two parallel randomized, double-masked phase 3 clinical trials (VIEW 1 and VIEW 2) on the efficacy and safety of VEGF Trap-Eye for the treatment of neovascular AMD were reported [42]. Phase I data demonstrated acceptable safety and tolerability of VEGF Trap-Eye in the treatment of neovascular AMD, and in Phase II study data, patients dosed in a similar fashion to the PrONTO trial demonstrated stabilization of their vision that was similar to previous studies of ranibizumab at 1 year. All dosing regimens of VEGF Trap-Eye, including 2 mg bimonthly, met the primary endpoint of noninferiority compared with monthly 0.5 mg ranibizumab with regard to the percentage of patients with maintenance (loss of <15 ETDRS letters) or improvement in vision. A greater mean improvement in visual acuity compared with monthly 0.5 mg ranibizumab at 1 year versus baseline represented the secondary endpoint of the study. In both the North American study (VIEW 1) and international study (VIEW 2), more than 95% of patients in each of the following VEGF Trap-Eye dosing groups achieved maintenance of vision compared with 94% of patients on monthly ranibizumab: 0.5 mg monthly, 2 mg monthly, and 2 mg every 2 months. In VIEW 1, patients on 2 mg monthly dosing achieved the secondary endpoint with a mean gain of 10.9 ETDRS letters compared with 8.1 for monthly ranibizumab (*P* < 0.01) [42]. 

In contrast to current anti-VEGF antibodies, which are rapidly cleared, the VEGF Trap-Eye is relatively degraded more slowly. Due to its high binding affinity and the ability to safely inject high doses into the eye, VEGF Trap-Eye may have longer duration of effect in the eye. Its adoption into clinical practice will depend on efficacy at 4- and 8-week intervals. If effective at 4- and 8-week intervals, VEGF Trap-Eye offers a competitive price advantage over ranibizumab and the opportunity to significantly reduce treatment burden on patients and physicians.

### 3.5. Future Therapies for Age-Related Macular Degeneration

AMD is a complex mechanism in which a variety of mediators are likely to be involved. Any of these could serve as a potential target for the treatment of ocular neovascularization. In preclinical and clinical studies, several targets have already been evaluated. For example, treatment regimes of a drug blocking the transduction of the signaling cascade within the cell (tyrosine kinase) and one inhibiting the further intracellular production of VEGF (small interfering RNA) might achieve a better visual outcome [43]. Yet the efficacy, safety, treatment interval, and cost of these treatments remain undetermined. But the increasing number of drugs affecting neovascular growth and leakage by different mechanisms will potentially allow various combination strategies.

## 4. Conclusion

Age-related macular degeneration (AMD) affects the many elderly population. Before the development of anti-VEGF, the diagnosis of neovascular AMD meant frequently loss of useful vision. But targeted anti-VEGF therapy has significantly improved the treatment of neovascular AMD. 

The appropriate method, dose, and types of combination therapy remain undetermined but randomized trials are currently continuing and will provide critical insight into the clinical applicability of new regimens. It hopefully can help in the treatment of resistant CNV with longer duration and less frequency between treatments.
